# Surveillance of salivary properties of pre-orthodontic patients in relation to age and sex

**DOI:** 10.1038/s41598-021-85861-8

**Published:** 2021-03-22

**Authors:** Isamu Kado, Ryo Kunimatsu, Yuki Yoshimi, Cynthia Concepcion Medina, Sakura Yamada, Kotaro Tanimoto

**Affiliations:** 1 Department of Orthodontics, Division of Oral Health and Development, Hiroshima University Hospital, 1-2-3 Kasumi, Minami-ku, Hiroshima city, 734-8554 Japan; 2 Department of Orthodontics, Graduate School of Biochemical and Health Sciences, Hiroshima University, 1-2-3 Kasumi, Minami-ku, Hiroshima city, 734-8554 Japan

**Keywords:** Epidemiology, Periodontitis

## Abstract

Saliva plays an important role in masticatory function and protection from dental caries. Although studies have been conducted on saliva properties, their results vary widely depending on population settings. Hence, this study was performed to evaluate the results of saliva properties in individuals who attended their first visit for orthodontic treatment. A total of 619 participants were included (387 females and 232 males; mean age: 14.6 years). We conducted oral examinations and saliva (stimulated) tests and evaluated the saliva flow rate, pH, and buffering capacity, along with bacterial culture. Saliva flow rate, pH, and buffering capacity were significantly higher in males than in females. However, the *Streptococcus mutans* score was significantly higher in females than in males even though oral hygiene was better in females. Significant positive correlations were found between age and saliva flow rate and *S. mutans* score. On the contrary, significant negative correlations were found between age and pH and buffering capacity. These results were similar to other studies where the target population was children or teenagers. Saliva properties of patients starting orthodontic treatment were almost the same as in populations of similar ages.

## Introduction

Saliva plays an important role in the maintenance of oral hygiene and aids in functions such as mastication and swallowing^1–3^. Generally, it is said that saliva production gradually decreases with age, and children can produce a greater volume of saliva than adults^4,5^. In previous studies, the salivary secretion volume in females was found to be lower than that in males for all age groups^6,7^. Furthermore, it is often assumed that saliva secretion capacity decreases with age^4,8^.

However, the results of studies on saliva flow rate vary depending on the researcher, the study population, and whether stimulated or unstimulated saliva was the target. With the absence of interference from influencing factors such as diurnal variation, stimulated saliva is more stable than unstimulated saliva. The saliva buffering (constant pH) capability is mainly due to the presence of bicarbonate, with additional contributions from phosphate and protein^9^. In terms of saliva pH and buffering capability, several studies have reported that the buffer effect was lower in females than in males, and was positively correlated with age^6,10^. Salivary secretion with this buffering capability, along with the oral cleaning effect, antibacterial actions, and supersaturated condition with calcium phosphate^11,12^ offers a protective role in dental caries. Furthermore, the higher the saliva flow rate, the lower the risk and prevalence of oral bacterial infectious diseases such as caries and periodontitis^13^.

*Streptococcus mutans* significantly contributes to dental caries by metabolising sucrose to lactic acid and by producing glycosyltransferase enzyme that establishes sticky biofilm chains. A positive correlation between the salivary count of *S. mutans* and caries morbidity was found in elementary school children; however, there were no differences according to sex and race^14^. *Lactobacillus spp.* are major bacteria of the lactic acid bacteria group^15^ and, similar to *S. mutans*^16^, is deeply involved in the development of dental caries by making the oral environment acidic*.* It has been proposed that there exists a tendency towards higher salivary counts of *Lactobacillus *spp. with advancing age^17^, and the *Lactobacillus *spp. count could be a useful method for prediction of caries in healthy individuals^18^.

Orthodontic treatment is common in managing malocclusions and patients who have undergone orthodontic treatment have a lower risk of caries in the future compared to those who have not^19^. However, we often experience iatrogenic disorders such as dental caries, white spot lesions, and gingivitis during treatment using orthodontic appliances^20–24^. Presently, orthodontists try to address these problems through specific guidance on prevention and oral care protocols for each patient and by further adjusting their visit intervals^25^.

Despite the availability of numerous studies about the properties of saliva, the data are scarce with respect to the population of patients starting orthodontic treatment. The goal of our research was to predict iatrogenic disorders that could be caused by bacterial infections during orthodontic treatment by performing specific tests at the first visit.

## Materials and methods

### Study design and ethics statement

This study used a cross-sectional design and was approved by the Independent Ethics Committee of Hiroshima University Hospital, Hiroshima, Japan (No. E-1039). Written informed consent was obtained from all the subjects following provision of a full explanation of the study. This study included subjects under the age of 18, in which case informed consent was obtained from the parent or legal guardian. All the methods were performed in accordance with the relevant guidelines and regulations.

### Study population

A total of 1,321 patients visited the Department of Orthodontics, Hiroshima University Hospital from April 2015 to December 2019. All patients received dental check-ups including plaque control record (PCR) performed by oral hygienists and underwent orthodontic examinations such as dental models, oral photos, lateral cephalometric records, and salivary tests at the first visit. We excluded patients from the final sample who lacked even one type of examination result, those who were on medicines such as antibiotics within 14 days leading up to the saliva test and who had congenital diseases including oligodontia. Thus, a final sample of 619 subjects (387 females, 232 males) was included. The subjects were classified by tooth alignment and malocclusion traits, such as arch length discrepancy (ALD), and antero-posterior, vertical, or horizontal aspects of malocclusion. Three orthodontists performed the analysis of the dental models and intraoral photos, and classified the malocclusions. With regard to ALD, subjects were classified according to the following criteria based on the amount of discrepancy; crowding < − 2 mm, − 2 mm < normal < 2 mm, and 2 mm < space arch. In the antero-posterior aspect of the malocclusion, subjects were classified as per the following criteria: with overjet; mandibular protrusion < 0 mm, 0 mm < normal < 4 mm, and 4 mm < maxillary protrusion. In the vertical aspect of the malocclusion, subjects were classified as per the following criteria: with overbite; open bite < 0 mm, 0 mm < normal < 4 mm, and 4 mm < deep bite. In the horizontal aspect of the malocclusion, subjects were classified according to premolar and molar crossbites.

### Saliva collection

Before sampling, subjects were asked to refrain from eating, drinking, smoking, and brushing their teeth for at least 2 h and to avoid using mouthwash and dentifrices. We collected the stimulated saliva samples while the subjects were seated on a dental chair in a relaxed state. Subjects were instructed to chew a tasteless gum contained in Checkbuf (Horiba, Kyoto, Japan). The saliva secreted in the first minute was spat out into a dental spittoon and stimulated saliva was collected in conical centrifuge tubes for 5 min by continued chewing. If the amount of saliva was less than 2 mL, the subjects continued to chew the tasteless gum until the total volume reached at least 2 mL. The collected saliva was immediately sent to a laboratory for subsequent examinations.

### Saliva examinations

Saliva flow rate, the volume of the saliva secreted per min (mL/min), was calculated. Saliva pH was measured with a digital pH metre. The buffering capability of saliva was estimated by the CAT21Buf Risk Test of Saliva (Morita Co., Osaka, Japan)^26^. The mixture of saliva and acid load liquid was vigorously shaken, and the final pH was measured. *S. mutans* and *Lactobacillus spp.* were cultured for 48 h at 37 °C using the CRT caries risk test (Ivoclar Vivadent, Tokyo, Japan) and semi-quantitative evaluation was performed in four stages (score 1, 2 < 10^5^ colony-forming units [CFU], score 3, 4 > 10^5^ CFU). *Candida albicans* was cultured in CHROMagar Candida medium (CHROMagar, Paris, France) for 48 h at 37 °C and semi-quantitative evaluation was performed in four stages (score 0: colony not found, score 1: colonies were found [at least single colony]).

### Statistical analysis

All data were processed with BellCurve for Excel (Society Survey Research Information Co., Ltd, Tokyo, Japan). Descriptive statistics were used for all variables including standard, means, and frequency. In the comparison between females and males, the data were compared using the Mann–Whitney *U* test and Fisher’s exact test. To consider the influence of age in each oral condition, the data were analysed using the Spearman’s rank correlation coefficient and Kruskal–Wallis test. The regression line in the regression analysis was drawn using the least-squares method.

## Results

The study subjects comprised 619 patients (387 females and 232 males; mean age 14.6 ± 9.9 years). Details of the distribution of the subjects in terms of age, sex, and malocclusions or dentofacial deformities are shown in Table 1.

**Table 1:** Baseline of subjects’ characteristics.

	Female			Male			All		
	n	n (%)	Median	n	n (%)	Median	n	n (%)	Median
**Age (years)**	387	63	11.8	232	37	10.7	619	100	11.0
**Malocclusion (arch length discrepancy)**									
Normal	43	61		28	39		71	100	
Space arch	65	56		52	44		117	100	
Crowding	279	65		152	35		431	100	
**Malocclusion (antero-posterior)**									
Normal	200	65		109	35		309	100	
Maxillary protrusion	133	63		77	37		210	100	
Mandibular protrusion	54	54		46	46		100	100	
**Malocclusion (vertical)**									
Normal	233	63		139	37		372	100	
Deep bite	85	55		69	45		154	100	
Open bite	69	74		24	26		93	100	
**Malocclusion (horizontal)**									
Normal	349	64		200	36		549	100	
Buccal cross-bite	27	52		25	48		52	100	
Lingual cross-bite	11	61		7	39		18	100	

### Oral hygiene status was better in females than males and the variation was large in the younger generation

**Figure 1:**
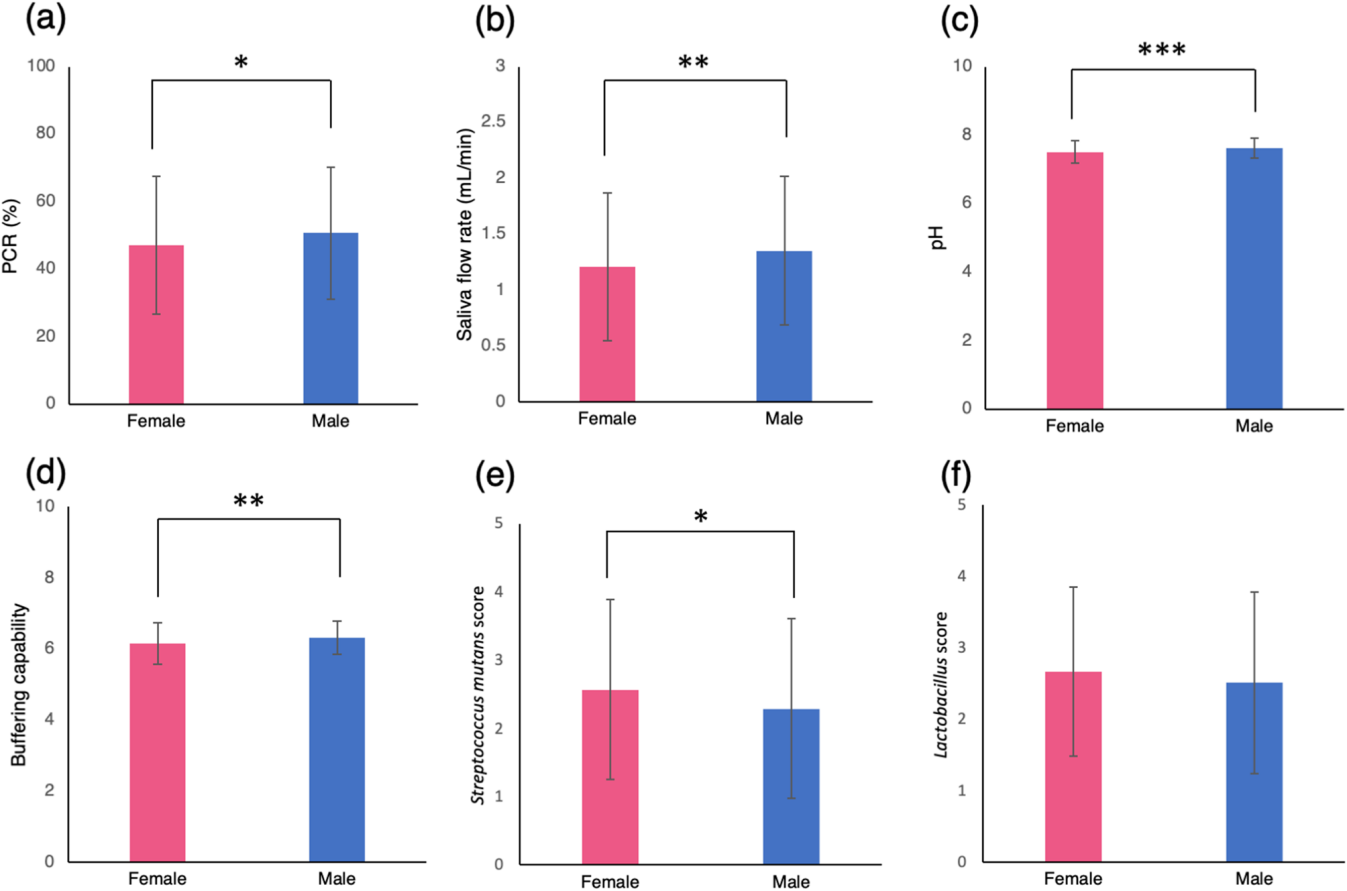
Comparison between females and males. Comparison of the average scores of (**a**) PCR, (**b**) Saliva flow rate, (**c**) pH, (**d**) Buffering capability, (**e**) *Streptococcus mutans* score, and (**f**) *Lactobacillus *spp. score between females (n = 387) and males (n = 232) using the Mann–Whitney *U* test. Significant differences are indicated as *p < 0.05, **p < 0.01 and ***p < 0.001. PCR, plaque control record.

**Figure 2:**
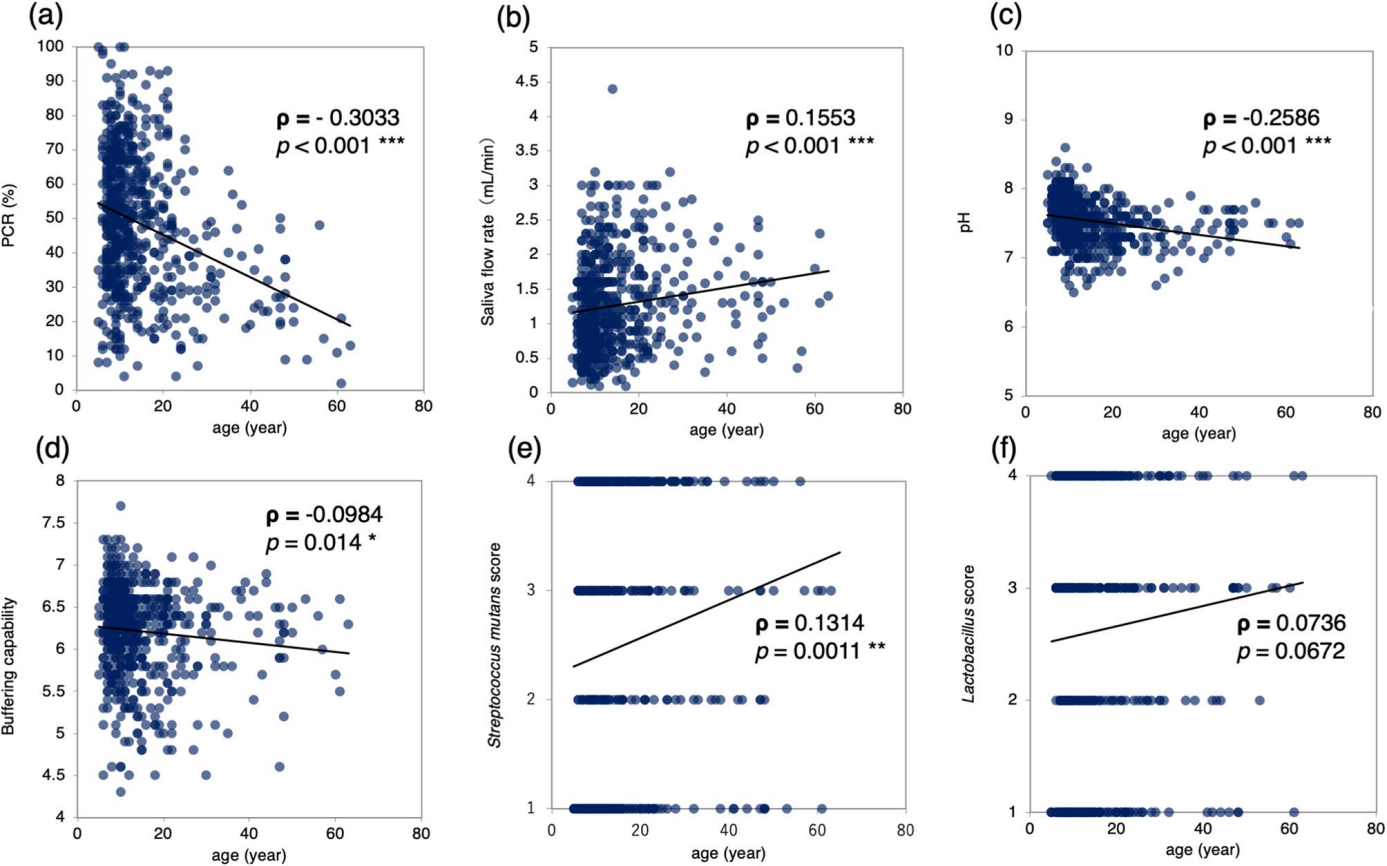
Correlation with age. Correlation between the scores of (**a**) PCR, (**b**) Saliva flow rate, (**c**) pH, (**d**) Buffering capability, (**e**) *Streptococcus mutans* score, and (**f**) *Lactobacillus spp.* score and subject’s age using the Spearman’s rank correlation coefficient. Each dot represents a subject, and the linear regression is shown by a solid line. The values of the correlation coefficient are shown as (ρ) and significant differences are indicated as *p < 0.05, **p < 0.01 and ***p < 0.001. PCR, plaque control record.

The average of PCR score was significantly higher (*p* = 0.0129) in males (50.7 ± 20.3%) than in females (47.1 ± 19.5%) (Fig. 1a). The overall mean PCR value was 48.44 ± 19.91% and ranged from 2 to 100% with large individual differences, especially among the younger generation. Spearman’s rank correlation coefficient revealed that PCR had a statistically significant negative correlation with age (*p* < 0.001) and the value of the correlation coefficient (ρ) was – 0.3033 (Fig. 2a). In other words, the older the subjects, the lower the PCR value.

### Males had less susceptible salivary properties to dental caries than female

Regarding the saliva flow rate, males (1.35 ± 0.67 mL/min) had a significantly higher (*p* = 0.0048) flow rate than females (1.21 ± 0.66 mL/min) (Fig. 1b). The overall mean saliva flow (mL/min) rate was 1.28 ± 0.66, ranging from 0.1 to 4.4. There was significant positive correlation with age (ρ = 0.1553, *p* < 0.001) (Fig. 2b). Salivary pH was significantly higher in male subjects (7.61 ± 0.29) than in females (7.50 ± 0.32) (*p* < 0.001) (Fig. 1c). In addition, salivary buffering capability was significantly higher in male subjects (6.32 ± 0.47) than in females (6.15 ± 0.58) (*p* = 0.0021) (Fig. 1d). There were large individual differences among the younger generations in salivary pH and buffering capability. Both salivary pH and buffering capability had significant negative correlations with age and the correlation coefficients were − 0.2586 (Fig. 2c) and − 0.0984, respectively (Fig. 2d).

### Gender difference in salivary *S. mutans*

**Figure 3:**
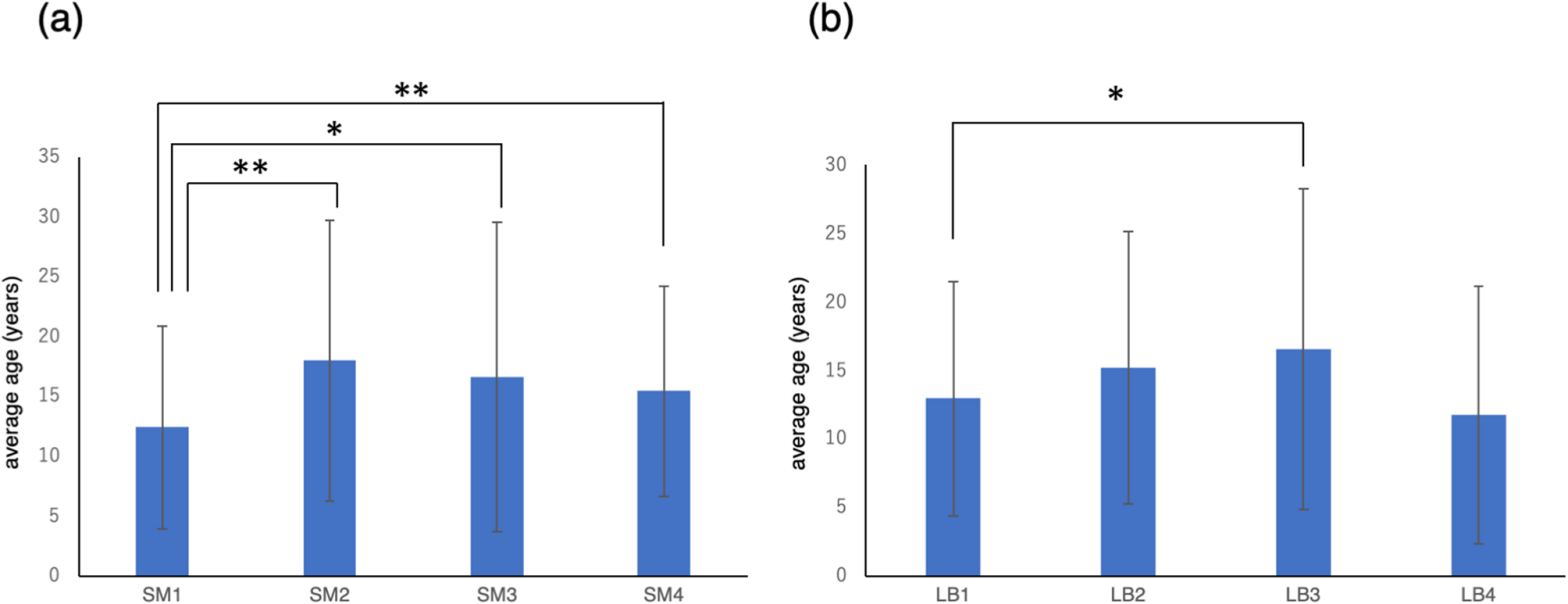
Evaluation of bacterial score and the average age. Comparison of the average age among the score of (**a**) *Streptococcus mutans* and (**b**) *Lactobacillus spp.* using the Kruskal–Wallis test. Significant differences are indicated as *p < 0.05, **p < 0.01 and ***p < 0.001.

Bacterial culture tests revealed that the *S. mutans* score was significantly higher in the female (2.57 ± 1.32) oral cavity than in the male (2.29 ± 1.32) oral cavity (*p* = 0.0152) (Fig. 1e). In relation to age, the *S. mutans* score showed significant positive correlation (ρ = 0.1314, *p* = 0.0011) (Fig. 2e). The average age for each *S. mutans* score was compared using the Kruskal–Wallis test, and the average age for score 1 was significantly lower than that for scores 2, 3, and 4 (Fig. 3a). Although *Lactobacillus spp.* showed the same tendency as *S. mutans,* there were no statistically significant differences based on sex. The average *Lactobacillus spp.* score for males was 2.51 ± 1.27 and for females was 2.67 ± 1.18 (Fig. 1f). The *Lactobacillus spp.* score showed a positive correlation with age but there was no significant difference (ρ = 0.0736) (Fig. 2f). The average age for each *Lactobacillus spp.* score was compared using the Kruskal–Wallis test, and the average age for score 1 was significantly lower (*p* = 0.0184) than that for score 3 (Fig. 3b). In terms of the relationship between the two genera of bacteria, a positive correlation was found (ρ = 0.3383). The results of the *Candida albicans* culture test showed no significant differences based on sex and age (ρ = − 0.0108). Positive correlations were found between *Candida albicans* and *S. mutans* (ρ = 0.2211) and *Lactobacillus spp.* (ρ = 0.1470) but the results were not significant.

## Discussion

Overall, males had poorer oral hygiene than females. However, their caries risk was lower based on the saliva flow rate, pH, buffering capability, and *S. mutans* count. We found that oral hygiene improved, and the saliva flow rate increased with age. In contrast, we found that pH, buffering capability, and bacterial counts were indicative of an increased caries risk.

### Oral hygiene status

In one study of young people, females had greater knowledge, a more positive attitude, healthier lifestyle choices, and favourable behaviour regarding oral hygiene than males^27^. There are a few reports that found no significant difference between females and males for oral hygiene during orthodontic treatment^28^. However, several investigations, using full fixed appliances, have proposed that females have better oral hygiene conditions than males during orthodontic treatment^29,30^. Kudirkaite et al. reported that the 16- to 18-year-old patient-group had better oral hygiene than a younger group^30^. In the present study, based on the PCR index, the results of oral hygiene conditions reflected previous reports in terms of sex and age differences (Figs. 1a, 2a). This could be because the age groups of the evaluated population were similar.

### Salivary properties

Saliva contains various antibacterial substances, namely lysozyme, lactoperoxidase, lactoferrin, immunoglobulin A, and mucin, in addition to water, electrolytes, and enzymes^31^. Thus, a reduced salivary flow might predispose to dental caries^32^. The specific timepoints were not uniform and we did not analyze the diurnal effects. Unstimulated salivary flow rate tends to increase from morning to night, much like diurnal variations in body temperature under control of the autonomic nervous system^33^. It has been reported that in females, unstimulated salivary flow rate and pH are higher during the ovulation period than during the menstrual period^33^. It was reported that saliva microbiome differed more between individuals than the diurnal variation^34^, and the pH differed greatly between day and night but does not fluctuate significantly among different timepoints during daytime^35^. Hence, we utilised a relatively stable stimulated saliva. Yeh et al*.* studied more than 1000 subjects (35 to 75 years) and reported a significant age-related decrease for both stimulated and unstimulated saliva. However, the trend was stronger in unstimulated saliva^4^. In elderly people over the age of 60, there were no sex differences in stimulated saliva flow rate^36^. In adults, the secretion of unstimulated saliva, as well as stimulated saliva, was significantly lower for females than for males. In females, the resting secretion rate negatively correlated with age. The buffer effect was also significantly lower in females and had a positive correlation with age. Therefore, with advancing age, females tend to catch up with males^6^. In a study conducted on various age groups, the secretion volume of both stimulated and unstimulated saliva secreted from the parotid gland was irrelevant regarding age^37^. Parvinen et al*.* reported that there was no significant influence of age on the stimulated saliva flow rate and the pH, and females had lower output than males^17^. On the contrary, other investigations on persons ranging in age from 21 to 93 years have insisted that the secretion rate of stimulated saliva increases with age^38^. Percival et al*.* have reported that the secretion rate of unstimulated saliva decreases with age, whereas stimulated saliva has no correlation with age^7^. In addition, they reported a tendency towards lower secretion rate for both unstimulated and stimulated saliva in females than in males, and found a negative correlation between the DMF index and the flow rates of unstimulated saliva, but no relationship to stimulated parotid saliva^7^. Evaluation of salivation volume of young children under 5 or 12 years reported that the flow rate of both unstimulated saliva and stimulated saliva showed no age- or sex-related differences^39,40^. Mazengo et al. reported that the mean salivary flow rate and buffer effect were slightly lower in females than males, and salivary flow rate was significantly lower in the 12-year-old group than the older age groups^10^. A study focusing on Japanese children aged from 5 to 12 years reported that at all ages, unstimulated saliva flow rate was more than double that of adults, and girls tended to have lower saliva volume than boys^5^. Taking into account that the average age of the population in this study was 14.6 years, the results that salivary flow rate, pH, and buffering capability were significantly higher in males than in females (Fig. 1b–d) are in agreement with the other studies. In addition, the significant positive correlation between saliva flow rate and ageing (Fig. 2b) was consistent with previous reports on populations similar to our study^41,42^.

### Bacteria cultural tests

The earlier *S. mutans* is detected in children, the higher the occurrence of dental caries^43^. In children between the ages of 2 and 5 years, *S. mutans* were detected in 43% while the detection frequency of *Lactobacillus spp.* was 11.5%. Despite the low correlations between the number of *S. mutans* or *Lactobacillus spp.* and diet in terms of sugar intake, there were highly significant correlations between caries incidence and the amount of bacteria in saliva^44^. The essential requirements, such as low pH, anaerobic environment, and access to carbohydrates, were necessary for sustained colonisation of *Lactobacillus spp.* in the human oral cavity^45^. *Lactobacillus spp.* are known to downregulate several virulence genes including acid tolerance genes such as aguD, and suppress growth and biofilm formation of *S. mutans*^46,47^. The ability of *Lactobacillus spp.* to inhibit *S. mutans* was more pronounced in clinical isolates from caries-free subjects^48^. The detection rate of *S. mutans* in saliva by the PCR method was higher in subjects aged 12 years old than in those aged 15 years old^49^. In our study, the significant increase in the number of *S. mutans* with age (Figs. 2e, 3a) seems to be due to the increase in the number of teeth associated with the eruption of permanent molars. Therefore, it cannot be concluded that caries risk is increasing just because of the increase in the number of cariogenic bacteria. Caries occurrence may be affected by diet rather than the prevalence of *S. mutans*^50^*. Candida albicans* correlated with advancing age by analysis of its protein in a study population with a mean age of 53.4 years^51^. A previous study focusing on children found no significant difference in the detection rate of *Candida* according to age or sex^52^. Likewise, in other saliva tests, *Candida* cultural tests show a concordance of the results due to population approximation.

In this study, we found that the salivary properties of pre-orthodontic patients had a very similar tendency to that of other studies that included healthy subjects. However, this study had the limitation of being a single time point cohort study. Despite this, our findings suggest that future studies of orthodontic patients may be applicable to other age-matched populations. In the future, we aim to build a preventive system to predict infectious diseases during orthodontic treatment and by expanding the population, adding other examinations such as microbiome analysis, and longer follow up.

## Conclusions

In conclusion, among pre-orthodontic patients, males had worse oral hygiene than females; however, males had a lower risk of caries. With ageing, improved oral hygiene and an increased saliva flow rate were noted.
